# The efficacy of aspirin to inhibit platelet aggregation in patients hospitalised with a severe infection: a multicentre, open-label, randomised controlled trial

**DOI:** 10.1007/s10238-023-01101-5

**Published:** 2023-06-09

**Authors:** Lieve Mees van Zijverden, Moya Henriëtte Schutte, Milou Cecilia Madsen, Tobias Nicolaas Bonten, Yvo Michiel Smulders, Chantal Maria Wiepjes, Jeske Joanna Katarina van Diemen, Abel Thijs

**Affiliations:** 1 Department of Internal Medicine, Amsterdam University Medical Centre Location Vrije Universiteit Amsterdam, De Boelelaan 1117, 1081HV Amsterdam, The Netherlands; 2 Department of Public Health and Primary Care, Leiden University Medical Centre, Albinusdreef 2, 2333ZA Leiden, The Netherlands

**Keywords:** Infection, Platelets, Platelet aggregation, Platelet activation, Thrombocytes, Aspirin, Acetylsalicylic acid

## Abstract

Patients with severe infection have an increased risk of cardiovascular events. A possible underlying mechanism is inflammation-induced platelet aggregation. We investigated whether hyperaggregation occurs during infection, and whether aspirin inhibits this. In this multicentre, open-label, randomised controlled trial, patients hospitalised due to acute infection were randomised to receive 10 days of aspirin treatment (80 mg 1dd or 40 mg 2dd) or no intervention (1:1:1 allocation). Measurements were performed during infection (T1; days 1–3), after intervention (T2; day 14) and without infection (T3; day > 90). The primary endpoint was platelet aggregation measured by the Platelet Function Analyzer® closure time (CT), and the secondary outcomes were serum and plasma thromboxane B2 (sTxB2 and pTxB2). Fifty-four patients (28 females) were included between January 2018 and December 2020. CT was 18% (95%CI 6;32) higher at T3 compared with T1 in the control group (*n* = 16), whereas sTxB2 and pTxB2 did not differ. Aspirin prolonged CT with 100% (95%CI 77; 127) from T1 to T2 in the intervention group (*n* = 38), while it increased with only 12% (95%CI 1;25) in controls. sTxB2 decreased with 95% (95%CI − 97; − 92) from T1 to T2, while it increased in the control group. pTxB2 was not affected compared with controls. Platelet aggregation is increased during severe infection, and this can be inhibited by aspirin. Optimisation of the treatment regimen may further diminish the persisting pTxB2 levels that point towards remaining platelet activity. This trial was registered on 13 April 2017 at EudraCT (2016-004303-32).

**Supplementary Information:**

The online version contains supplementary material available at 10.1007/s10238-023-01101-5.

## Introduction

Cardiovascular events can be triggered by a variety of common non-cardiovascular clinical conditions, particularly those who are associated with systemic inflammation. Acute systemic infection, like pneumonia, raises short-term risk of myocardial infarction approximately fivefold and risk of stroke up to eightfold [1–3]. This increased risk is mostly confined to the first 90 days after the onset of illness [4]; it seems to be highest within the first 14 days of admission and gradually reduces thereafter [1]. The causal mechanisms underlying this increased risk are unclear and so are targeted preventive strategies. There is some evidence suggesting that this increased risk could be mediated, at least partially, by platelet’s response to systemic inflammation [5]. For example, in patients with pneumonia, platelet aggregation is higher in those who subsequently develop myocardial infarction [6]. If hyperaggregation indeed occurs during infection, platelet inhibitors such as aspirin could possibly be used as a preventive strategy. Aspirin, or acetylsalicylic acid, is an effective drug for inhibiting platelets: It has been proven to decrease the risk of myocardial infarction, stroke or vascular death with 22% in secondary prevention of cardiovascular disease [7]. It works by irreversibly blocking cyclo-oxygenase (COX) in platelets and thereby preventing the formation of thromboxane A2, a potent inducer of platelet activation and aggregation [8]. However, aspirin’s antiplatelet activity varies between individual patients and conditions [9], and during infection, aspirin’s efficacy may be reduced due to increased platelet turnover. Also, several studies suggest that platelets are affected by a circadian rhythm, and that, therefore, certain dosing intervals of aspirin might be more effective than others in inhibiting platelets [10].

In view of these considerations, the objective of this trial was twofold: 1) to assess whether platelet hyperaggregation and activation occurs in patients hospitalised with an acute infection and 2) to assess aspirin’s efficacy to inhibit this during the course of the infection.

## Methods

### Study design and participants

This is a multicentre, open-label, randomised controlled trial and performed in four hospitals in Amsterdam and Amstelveen, the Netherlands (Amsterdam UMC, location VUmc, OLVG East location, OLVG West location, Amstelland Hospital). The study is registered at EudraCT (https://www.clinicaltrialsregister.eu/ctr-search/trial/2016-004303-32/NL), and the protocol was approved by the Medical Ethics Committee of the Amsterdam UMC. Written informed consent was obtained from all participants who were eligible and agreed to participate.

All patients who were newly admitted to the internal and pulmonary medicine wards of the above-named hospitals were screened for inclusion according to the following inclusion criteria: a primary clinical diagnosis of pneumonia, or an invasive urinary tract infection or a soft tissue infection; age above 18 years, hospitalisation for at least 24 h and having received at least one dose of antibiotics. Patients were excluded in case of active malignancy, a platelet count below 120*10^9^/L, known bleeding diathesis, and chronic use of immunosuppressants, antiplatelet therapy or other medication that influences haemostasis (for example NSAIDs, SSRIs and anti-coagulants). Because of logistic reasons and possible hyperaggregability due to COVID-19, patients with suspected or confirmed SARS-CoV-2 infection were also excluded.

### Randomisation and masking

After inclusion, patients were randomly allocated to either the intervention or the control group. Patients in the intervention group received aspirin (acetylsalicylic acid, non-enteric-coated), 80 mg to be taken orally once daily in the evening (at 8:00 PM) or 40 mg twice daily (at 8:00 AM and 8:00 PM) for 10 consecutive days (from day 4 of admission until day 14). The control group did not receive additional medication nor placebo. Randomisation was performed electronically in equal numbers (1:1:1) by the Castor Electronic Data Capture system (Ciwit B.V., the Netherlands). Patients and researchers were not blinded.

### Procedures

Blood sampling took place at three time points: prior to randomisation and during infection (T1; between days 1 and 3 of admission), after intervention (T2; at day 14 after admission) and without infection (T3; > 90 days after admission). The blood sampling was done between 8.00 and 10.00 AM, before the morning aspirin intake in the twice-daily regimen group. Compliance of aspirin intake was checked via self-reports in research diaries, as well as via pill counting. Patients were instructed to refrain from smoking 30 min prior to sampling, to refrain from caffeine 2 h prior to sampling and to have only a light breakfast. Blood samples were drawn from the antecubital vein through a 21-gauge needle, first into a precursor tube, then into a sodium citrate tube, a serum clot activator tube, a heparin tube and an EDTA tube.

The occurrence of cardiovascular events and other possible adverse events during the period of admission was continuously assessed by medical staff. If patients were discharged from the hospital, adverse events were monitored by the patients at home, and they were instructed to immediately report any event to the researchers.

### Outcomes

Our primary outcome was the Platelet Function Analyzer® closure time. The Platelet Function Analyzer® (PFA) measures platelet aggregation by simulating blood flow through an injured vessel. The closure time (CT) is the time needed for a blood plug to develop and occlude the cartridge. CT is inversely correlated with platelet aggregation; a prolonged CT means decreased aggregation [11]. Serum and plasma thromboxane B2 were secondary laboratory outcomes. Serum TxB2 is a reflection of maximum platelet activation, because it is measured after stimulating platelets to become fully activated *in vitro*. Plasma TxB2, on the other hand, is a reflection of platelet activity *in vivo*, because indomethacin is added to the blood sample to prevent activation of the platelets during the procedure of collecting blood. So, plasma TxB2 measurements reflect the degree of platelet activity as if the platelets are still situated inside the blood vessels [12]. Therefore, plasma TxB2 levels are relatively low. A disadvantage of measuring plasma TxB2 is that other cells, for example macrophages, sometimes produce small amounts TxB2 as well and, therefore, might affect the total plasma TxB2 level [12, 13]. For the purpose of measuring serum TxB2, platelets are stimulated whereafter constitutive secretion of TxB2 takes place. Consequently, TxB2 levels are relatively high, and therefore, the possible influence of TxB2 production by cells other than platelets is no longer relevant [12]. Most studies evaluating platelet activation only measure sTxB2. We chose to measure TxB2 both ways to compare, and improve our comprehension of, *in vitro* and *in vivo* platelet behaviour.

#### Platelet Function Analyzer® closure time

PFA-CT was assessed at the hospital where the patient was admitted. After discharge from the hospital, CT was assessed at Amsterdam UMC, location VUmc. In this hospital, the PFA-200 was used, the other hospitals used the PFA-100 (Siemens Healthineers, Erlangen, Germany). To measure CT, 800 µL of whole blood was added to the machine. A collagen/epinephrine test cartridge was used, which is sensitive to aspirin-mediated effects [14]. A CT greater than 300 s (the maximum) is reported as 301 for the purpose of data analysis.

#### Thromboxane B2

For the purpose of serum TxB2 measurement, the serum clot activator tube was incubated at 37 °C for 60 min immediately after blood withdrawal. Afterwards, the samples were centrifuged at 4000 × g for 10 min and stored at − 80 °C until analysis. To be able to measure plasma TxB2, the EDTA tube was immediately centrifuged after blood withdrawal at 4000 × g for 10 min and, after addition of indomethacin, stored at − 80 °C until analysis. All samples were analysed in the same laboratory by the use of enzyme immunoassay according to the manufacturer’s instructions (Item #10,004,023, Cayman Chemical, USA). Samples were analysed in duplicate to account for inter-assay variation, and the mean values were used in further analyses.

### Statistical analysis

Our sample size calculation was based on the question about aspirin’s efficacy of inhibiting platelet activity in aspirin-naïve patients with a severe infection. There are no data available in the current literature on the suggested size of aspirin’s effect. We, therefore, applied data from a pilot study conducted in 12 healthy aspirin-naive subjects [15]. Based on an expected effect size of 48% increase in CT after aspirin use, a power of 80% and a two-sided alpha level of 5%, we estimated that we needed to include 37 patients in the aspirin group. To register the natural course of platelet activity, we estimated that we needed to include 20 patients in the control group. We included + 5% in both the intervention (*n* = 40) and the control groups (*n* = 22) to account for potential dropout. Thus, in total, we estimated that 62 patients had to be included in the main trial.

**Fig. 1:**
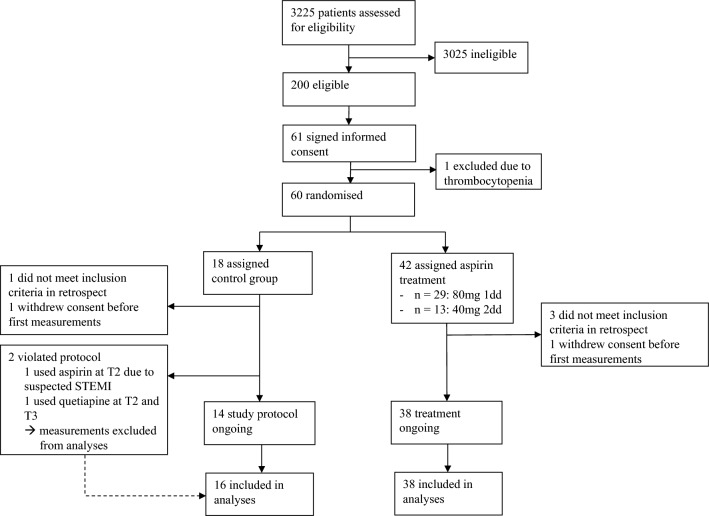
Flow diagram of the trial profile

All statistical analyses were performed using STATA® version 15.1. Baseline characteristics are expressed as mean with standard deviation for normally distributed continuous data and as median with interquartile range for non-normally distributed continuous data (Table 1). Before analysing the variables of our interest, measurements were excluded from the analysis in case of protocol violation (see Fig. 1). Data of sTxB2 in the intervention group and pTxB2 in both randomisation groups were not normally distributed, so a log transformation of all variables was performed. Hereafter, linear mixed models with measurements clustered within participants and with randomisation group as interaction term were performed to obtain changes with 95% confidence intervals of CT, sTxB2 and pTxB2 between the different time points. Results were back-transformed for presentation and expressed as percentage. Because of the non-normally distributed variables, the values at different time points were reported as geometric means with their 95% confidence intervals as STATA does not provide a standard deviation. All results were checked for sex and platelet count as a possible effect modifier. Figures for presentation were made using GraphPad Prism version 9.0.

**Table 1:** Baseline characteristics of the study population

	No intervention (*n* = 16)	Aspirin treatment (*n* = 38)
Age (years)	64 (52–69)	60 (46–72)
Female sex	8 (50%)	20 (53%)
*Smoking status*^*a*^		
Current smoker	4 (25%)	6 (16%)
Non smoker	12 (75%)	31 (82%)
BMI (kg/m^2^)	28 (25–31)	25 (23–28)
Systolic blood pressure (mmHg)	132 (120–153)	124 (109–136)
Diastolic blood pressure (mmHg)	76 (67–91)	75 (70–84)
Thrombocytes (× 10^9^/L)	216 (189–260)	224 (192–301)
CRP (mg/L)	131 (41–289)	175 (111–282)
Temperature (֯C)	38.2 (1.1)	38.2 (1.0)
*Infection type*		
Pneumonia	9 (56%)	21 (55%)
Positive blood culture^b^	1/8 (13%)	6/16 (38%)
Urinary tract infection	2 (13%)	12 (32%)
Positive blood culture^b^	1/2 (50%)	4/11 (45%)
Soft tissue infection	5 (31%)	5 (13%)
Positive blood culture^b^	0/4 (0%)	0/3 (0%)
*Aspirin dosage regimen*		
1dd 80 mg	–	27 (71%)
2dd 40 mg	–	11 (29%)

Data are presented as median (IQR), mean (SD) or *n* (%). BMI; body mass index. ^a^In the aspirin treatment group, data from one participant are missing. ^b^In some cases, no blood culture was collected

## Results

Patients were recruited between 11 January 2018 and 10 December 2020. Figure 1 shows the inclusion of patients in this study. Out of 3225 patients admitted with an acute infection, 200 were eligible, 61 signed informed consent and 54 were included in the final analyses. Unfortunately, due to the COVID-19 pandemic and the exclusion of patients with suspected or confirmed SARS-CoV-2 infection, the inclusion rate radically decreased. We, therefore, halted the twice-daily 40-mg group and focused on the regular dosage regimen of once-daily 80 mg. For analysis, we merged the two dosage groups together as the aspirin treatment group. Ultimately, there were 16 patients included in the control group and 38 in the aspirin group. Eleven (29%) participants followed the twice-daily aspirin regimen, and 27 (71%) used aspirin once daily. Baseline characteristics of the study population are shown in Table 1.

**Fig. 2:**
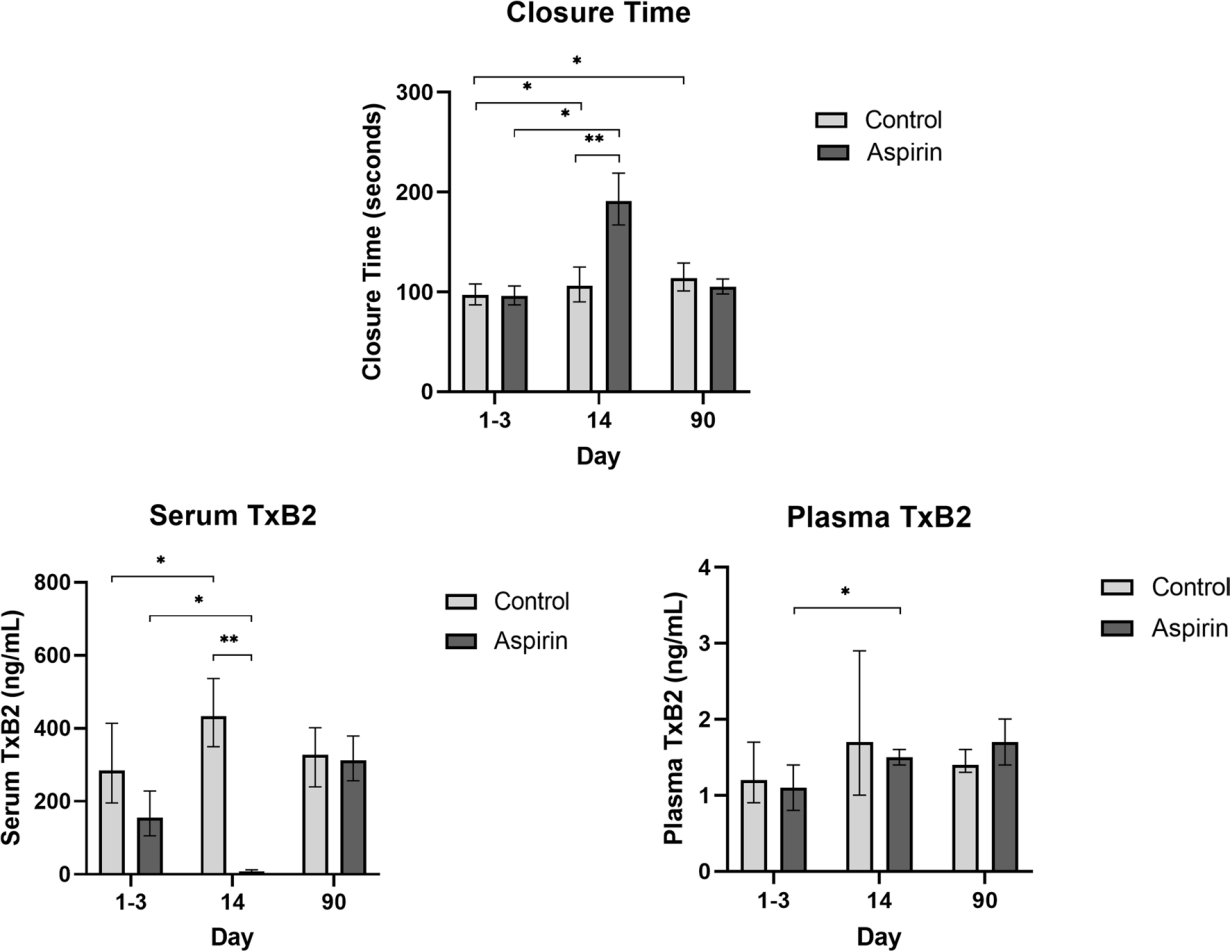
Closure time (CT), serum thromboxane (sTxB2) and plasma thromboxane (pTxB2) during infection (days 1–3, T1), after intervention (day 14, T2) and without infection (day > 90, T3) in the control and aspirin treatment group. Each bar represents the geometric mean with its 95% confidence intervals. Linear mixed models were used to analyse the percentage changes between different time points. * Significant change within group; ** the change in the aspirin group is significant compared to the change in the control group

Platelet function test results are described in Table 2 and visually presented in Fig. 2. Table 2 also displays the varying number of measurements available for each outcome at different time points, which is the results of some missing data. In the control group, CT was 18% (95%CI 6; 32) higher at T3 (without infection) compared with T1 (during infection). After recovery from infection at T3, sTxB2 was 15% (95%CI − 22; 70) higher than during infection at T1, but this was not significant. Lastly, a non-significant increase in pTxB2 of 19% (95%CI − 25; 89) at T3 compared with T1 was observed.

**Table 2:** Closure time (CT), serum thromboxane B2 (sTxB2) and plasma thromboxane B2 (pTxB2) at three different time points (during infection (days 1–3, T1), after intervention (day 14, T2) and without infection (day > 90, T3)) and the percentage change in relation to T1

		T1 (during infection, days 1–3)	T2 (after intervention, day 14)	T3 (without infection, day 90)	% change T1–T2	% change T1–T3
CT (s)	Control	97	106	114	+ 12%	+ 18%
		(87; 108)	(90; 125)	(101; 129)	(1; 25)*	(6; 32)*
		*n* = 16	*n* = 12	*n* = 12		
	Aspirin	96	191	105	** + 100%**	n.a
		(87; 106)	(167; 219)	(98; 113)	(77; 127)*	
		*n* = 35	*n* = 31	*n* = 30		
sTxB2 (ng/mL)	Control	284.6	433.1	327.2	+ 52%	+ 15%
		(195.7; 413.9)	(349.8; 536.2)	(239.6; 401.4)	(3; 125)*	(− 22; 70)
		*n* = 16	*n* = 12	*n* = 12		
	Aspirin	155.3	7.8	311.7	− **95%**	n.a
		(105.8; 228.1)	(5.0; 12.1)	(256.5; 378.9)	**(**− **97; **− 92)*	
		*n* = 36	*n* = 28	*n* = 30		
pTxB2 (ng/mL)	Control	1.2	1.7	1.4	+ 42%	+ 19%
		(0.9; 1.7)	(1.0; 2.9)	(1.3; 1.6)	(− 9; 123)	(− 25; 89)
		*n* = 16	*n* = 13	*n* = 12		
	Aspirin	1.1	1.5	1.7	+ 42%	n.a
		(0.8; 1.4)	(1.4; 1.6)	(1.4; 2.0)	(13; 80)*	
		*n* = 35	*n* = 31	*n* = 29		

Data are presented as geometric means (95% CI) or % (95% CI). Linear mixed models were used to analyse the percentage changes between different time points. The number of measurements per time point and outcome are described. * Significant change within group; bold text: Change in the aspirin group is significantly different from the change in the control group; n.a.: not analysed

In the intervention group, aspirin caused a prolongation of the CT of 100% (95%CI 77; 127) from T1 towards T2, which is significantly higher than the observed increase of only 12% (95%CI 1; 25) in the control group. For sTxB2, we also observed a significant effect of aspirin: The change in sTxB2 from T1 to T2 was − 95% (95%CI − 97; − 92), while in the control group, sTxB2 increased from T1 to T2 with 52% (95%CI 3; 125). The change in pTxB2 was comparable for both groups: + 42% (95%CI 13; 80) in the aspirin group and also + 42% (95%CI − 9; 123) in the control group. There were no significant differences between aspirin regimen groups (once-daily 80 mg and twice-daily 40 mg) for CT and pTxB2, but the effect of aspirin on sTxB2 decrease was slightly stronger in the once-daily aspirin regimen group compared with the twice-daily group (data not shown).

In the control group as well as the aspirin group, results were not influenced by sex. There were no associations between CT, sTxB2 or pTxB2 and platelet count at baseline, or change in platelet count throughout the study period. The platelet counts at different time points in the control group and the aspirin group are provided as supplementary information (SI 1).

## Discussion

The salient findings of this trial are as follows: 1) Platelet aggregation is increased during severe infection, and 2) aspirin is effective in inhibiting platelet aggregation and activation as measured by PFA-CT and serum TxB2, although no decrease in plasma TxB2 was observed.

The finding of increased platelet aggregation is in line with the results of several other studies, for example seen in patients with COVID-19 and other types of viral respiratory tract infection [16, 17]. Also, as mentioned before, platelet activation was higher in patients with pneumonia who later developed myocardial infarction [6]. Infection-induced platelet aggregation may, therefore, explain the increased incidence of adverse cardiovascular events during infection, which was also suggested by two previous studies [18, 19]. Three mechanisms may contribute to infection-induced platelet hyperaggregation. The first is activation of vascular endothelium during acute infection or sepsis: Endothelial cells adopt a pro-inflammatory and pro-coagulant phenotype under the influence of, amongst other factors, oxidative stress. This causes release of reactive oxygen species that stimulate platelet aggregation [20]. The second mechanism is increased platelet turnover during infection, which increases reactive immature platelets [21]. Lastly, several bacteria, such as *Staphylococcus aureus* and *Streptococcus* species, can directly activate platelets by receptor binding [5]. Surprisingly, we did not observe higher concentrations of sTxB2 and pTxB2 during acute infection. An explanation for this could be the fact that all patients had received at least one dose of antibiotics and possibly additional medication or fluids. There is some evidence, mostly from *in vitro* studies, that beta-lactam antibiotics can suppress thromboxane production and platelet aggregation after a prolonged use or in high doses [22, 23]. While it is unlikely that this could already have played a part after a maximum of 3 days of antibiotics use before the first blood collection, it cannot be entirely ruled out.

Our second finding is that aspirin is effective in inhibiting platelet aggregation and activation based on the observed prolongation of PFA-CT and decrease in sTxB2. This is consistent with studies in healthy subjects or patients with chronic cardiovascular disease [24, 25]. We now show that this is also true for severe infection. To our knowledge, our study is the first to assess the effect of aspirin by measuring both serum and plasma TxB2. Contrary to our expectations, a significant decrease in serum, but not plasma TxB2 was observed. This could imply that aspirin is effective in inhibiting thromboxane production after maximal activation of platelets *in vitro*, while at the same time, a steady minimal level of platelet cyclo-oxygenase activity remains present in the patient’s bloodstream. Should our results translate in clinical endpoints, it might be feasible to try to improve aspirin's dosage regimen. Besides a higher dose, a shorter dosage interval could be considered. This has also been suggested by some studies that found an effect of circadian rhythm on platelets and a better inhibition with a twice-daily aspirin regimen [10]. Another explanation for the remaining levels of plasma TxB2 could be the production of pTxB2 by a different source: While platelets account for the majority of thromboxane production, it is also produced by macrophages, lungs and kidneys. However, it is thought that this production by cells other than platelets mostly occurs during local organ dysfunction [12, 13].

Despite the maintained pTxB2 levels, the reduced level of platelet aggregation and activatability after aspirin intake during acute infection could be of clinical relevance in terms of cardiovascular disease prevention. Similar promising results have been observed in other clinical studies on aspirin therapy during infection. For example chronic aspirin use is associated with a lower occurrence of cardiovascular events and a lower mortality rate in patients hospitalised for infection [26]. A randomised controlled trial in patients with an infection reported that aspirin therapy for a month reduced the risk of acute coronary syndrome with 9%, without a significant increase in bleeding [27]. Another randomised controlled study that was conducted with septic patients found a reduction in biomarkers of endothelial injury and platelet consumption in those who were treated with the platelet-inhibiting medication eptifibatide along with iloprost, as compared to patients receiving placebo [28]. These findings further emphasise the possible feasibility of platelet inhibition therapy during acute infection.

Strengths of this trial include the use of three different approaches to measure platelet aggregation and activation, the strict exclusion criteria regarding medication use and the standardisation of blood sampling and laboratory procedures. An important limitation is the early termination of the study, resulting in a small sample size and the inability to compare the two aspirin dosage regimens. However, the observation of changes in platelet aggregation despite the small sample size strengthens our hypothesis. Future research is needed to establish the effects of aspirin administration on clinical endpoints. Therefore, we designed the AS-CAP trial. This is a multicentre, randomised controlled trial in which clinical endpoints will be assessed in patients hospitalised with a severe infection.

In conclusion, this multicentre, open-label, randomised controlled trial in patients without a history of cardiovascular disease provides new insights into platelet activation and inhibition during infection. Our study suggests that platelet aggregation is increased in patients with a severe infection, and that aspirin inhibits this process. However, no inhibition of plasma TxB2 was achieved, which means that platelet cyclo-oxygenase residual activity might remain present despite aspirin use. Altogether, we can conclude that aspirin is a promising drug in the prevention of hyperaggregation due to acute infection.

### Supplementary Information

Below is the link to the electronic supplementary material.

Supplementary file1 (DOCX 12 KB)

## Data Availability

The metadata of this study will be added to the catalogue of the Dutch CardioVascular Alliance (DCVA; http://catalogue.dcvalliance.nl/) upon publication. The deidentified source data can be requested via the catalogues website and will be provided by the corresponding author after approval of the proposal. The study protocol is available via EudraCT (https://www.clinicaltrialsregister.eu/ctr-search/trial/2016-004303-32/NL).

## References

[1] Smeeth L, Thomas SL, Hall AJ, Hubbard R, Farrington P, Vallance P (2004). Risk of myocardial infarction and stroke after acute infection or vaccination. N Engl J Med.

[2] Corrales-Medina VF, Serpa J, Rueda AM (2009). Acute bacterial pneumonia is associated with the occurrence of acute coronary syndromes. Medicine (Baltimore).

[3] Musher DM, Abers MS, Corrales-Medina VF (2019). Acute infection and myocardial infarction. Reply N Engl J Med.

[4] Elkind MS, Carty CL, O’Meara ES (2011). Hospitalization for infection and risk of acute ischemic stroke: the cardiovascular health study. Stroke.

[5] Feldman C, Anderson R (2020). Platelets and their role in the pathogenesis of cardiovascular events in patients with community-acquired pneumonia. Front Immunol.

[6] Cangemi R, Casciaro M, Rossi E (2014). Platelet activation is associated with myocardial infarction in patients with pneumonia. J Am Coll Cardiol.

[7] Antithrombotic Trialists’ Collaboration (2002). Collaborative meta-analysis of randomised trials of antiplatelet therapy for prevention of death, myocardial infarction, and stroke in high risk patients. BMJ.

[8] Schrör K (1997). Aspirin and platelets: the antiplatelet action of aspirin and its role in thrombosis treatment and prophylaxis. Semin Thromb Hemost.

[9] Pappas JM, Westengard JC, Bull BS (1994). Population variability in the effect of aspirin on platelet function. Implications for clinical trials and therapy. Arch Pathol Lab Med.

[10] Buurma M, van Diemen JJK, Thijs A, Numans ME, Bonten TN (2019). Circadian rhythm of cardiovascular disease: the potential of chronotherapy with aspirin. Front Cardiovasc Med.

[11] Hayward CP, Harrison P, Cattaneo M, Ortel TL, Rao AK (2006). Platelet physiology subcommittee of the Scientific and Standardization Committee of the International Society on Thrombosis and Haemostasis. platelet function analyzer (PFA)-100 closure time in the evaluation of platelet disorders and platelet function. J Thromb Haemost.

[12] Patrono C, Rocca B (2019). Measurement of thromboxane biosynthesis in health and disease. Front Pharmacol.

[13] Remuzzi G, FitzGerald GA, Patrono C (1992). Thromboxane synthesis and action within the kidney. Kidney Int.

[14] Crescente M, Di Castelnuovo A, Iacoviello L, de Gaetano G, Cerletti C (2008). PFA-100 closure time to predict cardiovascular events in aspirin-treated cardiovascular patients: a meta-analysis of 19 studies comprising 3,003 patients. Thromb Haemost.

[15] Racca C, van Diemen JJK, Fuijkschot WW (2019). Aspirin intake in the morning is associated with suboptimal platelet inhibition, as measured by serum thromboxane B(2,) during infarct-prone early-morning hours. Platelets.

[16] Kreutz RP, Bliden KP, Tantry US, Gurbel PA (2005). Viral respiratory tract infections increase platelet reactivity and activation: an explanation for the higher rates of myocardial infarction and stroke during viral illness. J Thromb Haemost.

[17] Bongiovanni D, Klug M, Lazareva O (2021). SARS-CoV-2 infection is associated with a pro-thrombotic platelet phenotype. Cell Death Dis.

[18] Puurunen MK, Hwang SJ, Larson MG (2018). ADP platelet hyperreactivity predicts cardiovascular disease in the FHS (Framingham Heart Study). J Am Heart Assoc.

[19] Vazquez-Santiago M, Vilalta N, Cuevas B (2018). Short closure time values in PFA-100(R) are related to venous thrombotic risk. Results from the RETROVE Study. Thromb Res.

[20] Joffre J, Hellman J (2021). Oxidative stress and endothelial dysfunction in sepsis and acute inflammation. Antioxid Redox Signal.

[21] Liu QH, Song MY, Yang BX, Xia RX (2017). Clinical significance of measuring reticulated platelets in infectious diseases. Medicine (Baltimore).

[22] Burroughs SF, Johnson GJ (1990). Beta-lactam antibiotic-induced platelet dysfunction: evidence for irreversible inhibition of platelet activation in vitro and in vivo after prolonged exposure to penicillin. Blood.

[23] Mihara S, Fujimoto M, Okabayashi T (1986). Suppression by beta-lactam antibiotics of thromboxane A2 generation and arachidonic acid release in rabbit platelets in vitro. Thromb Res.

[24] Thygesen SS, Hvas AM, Kristensen SD (2007). Low-dose aspirin inhibits serum thromboxane B2 generation more than 99 percent. Blood.

[25] Kovacs EG, Katona E, Bereczky Z (2014). Evaluation of laboratory methods routinely used to detect the effect of aspirin against new reference methods. Thromb Res.

[26] Falcone M, Russo A, Cangemi R (2015). Lower mortality rate in elderly patients with community-onset pneumonia on treatment with aspirin. J Am Heart Assoc.

[27] Oz F, Gul S, Kaya MG (2013). Does aspirin use prevent acute coronary syndrome in patients with pneumonia: multicenter prospective randomized trial. Coron Artery Dis.

[28] Berthelsen RE, Ostrowski SR, Bestle MH, Johansson PI (2019). Co-administration of iloprost and eptifibatide in septic shock (CO-ILEPSS)-a randomised, controlled, double-blind investigator-initiated trial investigating safety and efficacy. Crit Care.

