# Supplementary material for: The efficacy of aspirin to inhibit platelet aggregation in patients hospitalised with a severe infection: a multicentre, open-label, randomised controlled trial
Source: Clin Exp Med. 2023 Jun 9;23(7):3501–8. doi: 10.1007/s10238-023-01101-5 (PMC10250844; doi:10.1007/s10238-023-01101-5)
Supplement: Supplementary file 1 — Supplementary file1 (DOCX 12 KB) [file 10238_2023_1101_MOESM1_ESM.docx]

**Supplementary Information**

|  |  | T1 (during infection,  day 1-3) | T2 (after intervention, day 14) | T3 (without infection,  day 90) | % change T1 to T2 | % change T1 to T3 |
| --- | --- | --- | --- | --- | --- | --- |
| Platelet count (x10^9^/L) | Control | 258  (223; 297)  *n=16* | 399  (343; 466)  *n=12* | 257  (228; 289)  *n=12* | +55%  (39; 74)* | +2%  (-9; 14) |
|  | Aspirin | 252  (219; 289)  *n=36* | 463  (402; 535)  *n=31* | 242  (214; 274)  *n=30* | +84%  (57; 116)* | -4%  (-19; 13) |

**SI 1.** Platelet count at three different time points (during infection (day 1-3, T1), after intervention (day 14, T2) and without infection (day >90, T3)) and the percentage change in relation to T1.

Data are presented as geometric means (95% CI) or % (95% CI). Linear mixed models were used to analyse the percentage changes between different time points. The number of measurements per time point and outcome are described. * Significant change within group.
